# A Mixed‐Methods Exploration of the Quality of Care and Care Needs for Asian Children and Young Adults With Serious Long‐Term Conditions

**DOI:** 10.1111/hex.70892

**Published:** 2026-10-07

**Authors:** Shih‐Chun Lin, Mei‐Chih Huang, Hsiang‐Ying Lu, Ying‐Chu Chen, Chao‐Neng Cheng, Frank Leigh Lu, Jong‐Hau Hsu

**Affiliations:** ^1^ School of Nursing, College of Nursing Kaohsiung Medical University Kaohsiung Taiwan; ^2^ Center for Long‐Term Care Research Kaohsiung Medical University Kaohsiung Taiwan; ^3^ National Tainan Junior College of Nursing Tainan Taiwan; ^4^ Department of Nursing, College of Medicine National Cheng Kung University Tainan Taiwan; ^5^ Department of Nursing National Cheng Kung University Hospital Tainan Taiwan; ^6^ Department of Paediatrics, National Cheng Kung University Hospital, College of Medicine National Cheng Kung University Tainan Taiwan; ^7^ Division of Pediatric Pulmonology and Critical Care Medicine, Department of Paediatrics, National Taiwan University Children's Hospital National Taiwan University Hospital Taipei Taiwan; ^8^ College of Medicine National Taiwan University Taipei Taiwan; ^9^ Department of Pediatrics, School of Medicine, College of Medicine Kaohsiung Medical University Kaohsiung Taiwan; ^10^ Department of Pediatrics, Kaohsiung Medical University Hospital Kaohsiung Medical University Kaohsiung Taiwan

**Keywords:** caregivers, family‐centred care, palliative care, patient‐reported outcome measures, paediatric nursing, quality of care

## Abstract

**Background or Context:**

Children and young adults with serious long‐term conditions frequently have multidimensional palliative care needs before specialist palliative care referral. However, evidence from Asian populations regarding reported care outcomes and the care needs of children, young adults, and their families remains limited.

**Objective:**

To compare patient‐ or proxy‐reported outcomes across levels of professionally assessed palliative care needs and explore felt and unmet care needs from the perspectives of family caregivers and healthcare professionals.

**Design:**

A convergent mixed‐methods design.

**Setting and Participants:**

Participants included 88 caregiver‐patient dyads from three medical centres in Taiwan. Patients were aged 1–25 years, had Paediatric Palliative Screening Scale (PaPaS) scores ≥ 15, and had not received specialist palliative care. Twelve family caregivers and 14 healthcare professionals were interviewed.

**Main Outcome Measures:**

PaPaS Scale, the African Children's Palliative Care Outcome Scale (C‐POS), and semi‐structured interviews.

**Results:**

Participants with PaPaS scores ≥ 20 had poorer outcomes for pain, crying, and playing than those scoring 15–19, and their caregivers had greater difficulty sharing their feelings (all *p* < 0.05; standardised mean differences, 0.35–0.55). Maternal employment status was associated with total C‐POS scores (*p* = 0.005). Four themes described seeking evolving care guidance, integrating care into family routines, sharing enduring caregiving responsibilities, and balancing present living with future preparation. Integration showed that greater normative needs were accompanied by challenges in symptom recognition, everyday participation, and caregiver emotional communication. Although caregivers generally reported receiving adequate support, the qualitative findings indicated that support was most useful when translated into coordinated and practical care plans tailored to family routines and readiness.

**Conclusion:**

Integrating professional assessments, reported outcomes, and caregiver and healthcare professional experiences identified complementary care needs. The findings support coordinated child‐ and family‐centred care that addresses evolving symptoms, everyday routines, caregiver burden, emotional communication, and families’ changing time orientations before referral to specialist palliative care.

**Patient or Public Contribution:**

A parent expert reviewed study materials and recruitment strategies and contributed to interpretation of the findings.

## 1 Introduction

Children and young adults with serious long‐term conditions face substantial risks of health complications and premature death. Family‐centred coordinated care, including specialist palliative care, can improve continuity across care settings and outcomes for both patients and their families [1]. As the number of children and young adults living with these conditions continues to increase worldwide, paediatric and palliative care services must be prepared to respond to their multidimensional needs [2, 3]. However, access to paediatric palliative care remains uneven across the Asia‐Pacific region. Resource and workforce limitations, insufficient policy support, and limited professional knowledge can delay its integration [4, 5]. Moreover, much of the available evidence has been generated among children and families already receiving specialist palliative care [1, 6], leaving the reported outcomes and felt or unmet care needs of those not yet referred less well understood. This evidence gap is particularly important in Asian healthcare contexts, which remain underrepresented in paediatric palliative care research. Examining care outcomes and needs before specialist referral may identify concerns that remain unrecognised within existing services and inform earlier, needs‐based support and referral.

Care needs can be understood from the normative, felt, and unmet perspectives. Normative needs arise when a person's condition or care falls short of a professionally defined standard, whereas felt needs refer to concerns recognised by children or caregivers themselves [7]. Unmet needs occur when identified or felt needs are not adequately addressed by available health and social care services [7, 8]. Although these dimensions may overlap, professionally assessed needs do not necessarily correspond with families’ experiences, and concerns important to families may not be detected through standardised clinical assessments.

The Paediatric Palliative Screening Scale (PaPaS Scale) supports healthcare professionals in identifying children who may benefit from palliative care; a score of ≥ 15 indicates that palliative care may be initiated [9, 10]. Nevertheless, some children with evident palliative care needs may still be considered ‘too well’ for referral [11]. Moreover, the PaPaS Scale identifies the level of normative palliative care need but provides limited information about which aspects of care are being adequately addressed.

The African Children's Palliative Outcome Scale (C‐POS) complements this professional assessment by capturing patient‐ or proxy‐reported outcomes across physical, emotional, social, communication, and family domains [12, 13]. Comparing C‐POS outcomes across PaPaS‐defined levels of needs can reveal areas of alignment or discrepancy between professionally assessed needs and the experiences reported by children and families. However, standardised measures do not fully explain how caregivers recognise, prioritise, and manage these concerns in everyday life. Therefore, qualitative exploration is required to contextualise quantitative patterns and identify felt or unmet needs that may not be captured by either screening or outcome measures. Accordingly, this convergent mixed‐methods study aimed to compare patient‐ or proxy‐reported outcomes across levels of professionally assessed palliative care needs and to explore care needs from the complementary perspectives of family caregivers and healthcare professionals.

## 2 Materials and Methods

### 2.1 Design

A convergent mixed‐methods design was used to develop a comprehensive understanding of the care needs of children and young adults with serious long‐term conditions who had not been referred to specialist palliative care. The study comprised the following:

1. A multicenter, observational, and cross‐sectional survey between April 2021 and August 2024.
2. Semi‐structured interviews with family caregivers and healthcare professionals between May 2020 and September 2023.

The components were conducted over overlapping periods, analysed separately, and integrated during interpretation. Quantitative analysis compared patient‐ or proxy‐reported C‐POS outcomes across levels of normative palliative care needs identified using the PaPaS Scale. The qualitative analysis explored family caregivers’ felt and unmet needs, together with healthcare professionals’ perspectives on the care and support required by children, young adults, and their families. Integration examined the convergence, complementarity, and divergence between professionally assessed needs, reported outcomes, and caregivers’ experiences, providing a more comprehensive understanding than either method alone. As a qualitatively led study, the qualitative component was reported with reference to the Reflexive Thematic Analysis Reporting Guidelines (RTARG) [14].

### 2.2 Participants

Paediatric patients who took part in the survey were deemed eligible if he/she (1) was aged 1–25 and (2) had PaPaS scores ≥ 15 [10]. Patients were excluded if they had received any national health insurance‐covered specialist palliative care services, such as palliative shared care, inpatient hospice, and palliative home care [2].

The eligibility criteria for the qualitative interviews included family caregivers aged 20 and above who had provided care for a child aged 1‐25 with serious long‐term conditions and professionals who had provided hands‐on or advisory palliative care for children within the past year. This study was conducted at three medical centres in Taiwan.

### 2.3 Recruitment

Before approaching potential participants, the research team assessed the PaPaS scores by reviewing patient records and consulting with other healthcare team members. They selected responses that best described the patients. A total of ninety‐three children and young adults were approached by trained research assistants at three medical centres. Of these, 88 were recruited for the study and completed the questionnaires, resulting in a response rate of 94.6% (two declined due to illness and three did not provide consent). Recruitment for the qualitative interviews was facilitated through professional networks, posters, social media platforms, and snowball sampling techniques. Recruitment ceased when the dataset was judged to provide sufficient depth, diversity, and relevance to address the research questions. This judgement was made iteratively during data collection and analysis, considering the specificity of the participant group, the richness of the interviews, and the extent to which later interviews elaborated rather than substantially altered the developed themes.

### 2.4 Data Collection: Survey

Family caregivers provided demographic information about themselves and their children. The PaPaS Scale, a validated tool for identifying children and young adults in need of palliative care, was used [9, 10, 15]. This scale consists of 11 questions addressing aspects including disease trajectory, treatment outcomes, symptom burden, healthcare preferences, and life expectancy. The responses can yield binary answers or fit into four ordinal categories. In this study, patients with high palliative care needs were defined as those with PaPaS scores of 20 or higher, whereas those with moderate palliative care needs had scores ranging from 15 to 19.

The C‐POS [16], a 12‐item instrument measuring quality of care, was employed based on input from children/young adults and caregivers. Responses are scored on a scale ranging from 0 (never) to 5 (all of the time), focusing on experiences from yesterday and today. The tool includes seven items for children/young adults and five for family caregivers, demonstrating strong validity and acceptability [17]. The original English C‐POS was translated into Mandarin using a forward‐backward process, led by two independent Mandarin researchers. The first output, built after comparison and agreement between the translators, was sent to two independent translators, a palliative care specialist, and a paediatric nurse. Minor changes were discussed and implemented by the English and Mandarin research teams. The revised Mandarin C‐POS was reviewed by 17 experts, including paediatricians, nurse practitioners, a psychotherapist, and a case manager, using a nine‐point scale to assess item appropriateness. The average scores ranged from 7.75 to 8.77. The C‐POS data were collected through face‐to‐face interactions with trained research assistants, as recommended by Coombes et al. [12].

### 2.5 Interviews

Data from the semi‐structured interviews were audio‐recorded with the participants’ permission. Research groups with methodological and clinical expertise reviewed the interview guide. The participants were asked to describe their experiences with caring for children and young adults with serious long‐term conditions; which services they believe need to be coordinated; any conflicts that may arise among patients, family caregivers, and professionals; how they managed these conflicts; the process of caregiving and its impact on the family; and any further insights into the needs of family caregivers. Interviews, led by the first author, lasted approximately 1 h and took place in the participants’ preferred quiet and private locations.

### 2.6 Analysis: Survey

The C‐POS scores for patients with a PaPaS score of 15‐19 and those with a score of ≥ 20 were compared using chi‐square or Mann‐Whitney U test for categorical or continuous variables, respectively. For the analysis, reverse scoring was applied to negatively worded items 3, 9, 10, 11, and 12 in the C‐POS total scores. Because the analyses were exploratory, no adjustment for multiple comparisons was applied. For comparisons of individual C‐POS items between the PaPaS groups, unadjusted p‐values and standardised mean differences were reported and interpreted cautiously. In the current sample, the child‐related items 1‐7 demonstrated acceptable internal consistency (McDonald's ω = 0.72, 95% CI [0.64, 0.79]), whereas the caregiver‐related items 8‐12 showed lower internal consistency (McDonald's ω = 0.62, 95% CI [0.47, 0.74]).

### 2.7 Interviews

Interview data were analysed using reflexive thematic analysis [14], informed by social constructionism and interpretivism. The first author, an experienced qualitative researcher and paediatric nurse, conducted data familiarisation, inductive coding, and theme development. The codes and themes were iteratively reviewed and refined with attention paid to their underlying relationships. The researcher's clinical background facilitated rapport and sensitivity to participants’ concerns but may also have influenced the interpretation of health‐service and caregiving issues. Reflexive notes were maintained to examine assumptions, emotional responses, and emerging interpretations. Regular discussions within the multidisciplinary research team, together with peer debriefing and member checking, enhanced the credibility of the analysis. Thick descriptions of the study context, participants, and findings supported transferability by enabling readers to assess their potential applicability to other contexts, including chronic illness care and relationships between professionals and families [18].

### 2.8 Mixed‐Methods Integration

Quantitative and qualitative data were analysed separately and concurrently. Formal integration occurred during interpretation by comparing quantitative associations with qualitative themes and examining areas of convergence, complementarity, and divergence. The qualitative findings contextualised the quantitative patterns and identified care needs that were not captured by the standardised measures. The integrated findings were subsequently used to develop an integrated interpretation.

### 2.9 Patient or Public Contribution

A mother of two children diagnosed with serious long‐term conditions participated in the study as a parent expert. With over 20 years of experience in palliative care services, she monitored the study's progress, reviewed interview guides and consent forms, contributed to participant recruitment strategies, provided insights into the findings, and assisted with support materials.

## 3 Results

### 3.1 C‐POS Outcomes Across Levels of Normative Palliative Care Needs

A total of 88 children and young adults with serious long‐term conditions participated in this study. The total C‐POS score did not differ significantly according to children or family characteristics, except for maternal employment. Maternal employment status was associated with total C‐POS scores, with more favourable scores observed among children and young adults whose mothers were employed (Table 1). Compared with children and young adults with PaPaS scores of 15‐19, those with scores ≥ 20 reported poorer outcomes for pain, crying, and playing. Their caregivers also reported greater difficulty in sharing their feelings. The standardised mean differences for these statistically significant comparisons ranged from 0.35 to 0.55 (all *p* < 0.05; Table 2). Child‐related C‐POS scores did not differ significantly between self‐reports (n = 43) and caregiver proxy reports (n = 45; *p* = 0.21).

**Table 1:** Comparison of C‐POS outcomes based on demographic and clinical features.

	Total	C‐POS			*p*‐value
		Mean ± SD			
Total	88	17.82 ± 8.45			
**Children/young adults characteristics**					
**Gender**						
Females	34	18.91 ± 9.21			0.34
Males	54	17.13 ± 7.95			
**Age at interview, years**						
1–12	34	17.71 ± 7.12			0.92
13‐25	54	17.89 ± 9.25			
**Seniority**						
Eldest	49	17.80 ± 7.80			1.00
Second eldest	26	17.92 ± 8.46			
Third youngest and below	13	17.69 ± 11.21			
**Family characteristics**						
**Primary caregiver**						
Father	14	19.93 ± 8.15			0.60
Mother	63	17.37 ± 8.13			
Other	11	17.73 ± 10.85			
**Family marital status, *n* (%)**					
Married/partnered	65	17.74 ± 8.81			0.28
Separated/divorced/single	23	19.48 ± 7.27			
**Additional siblings in the home**					
None	41	18.37 ± 7.95			0.57
1	34	16.65 ± 8.21			
2+	13	19.15 ± 10.75			
**Siblings disabled or aged** ≤ **3 years**					
None	10	18.90 ± 10.09			0.67
1+	78	17.68 ± 8.28			
**Religious affiliation**						
Atheist	18	18.72 ± 10.16			0.76
Folk beliefs/Buddhism/Taoism	61	17.38 ± 8.05			
Other	9	19.00 ± 8.14			
**Middle‐low‐/low‐income household**	13	18.31 ± 9.16			0.82
	75	17.73 ± 8.39			
**Care expenses**						
Very heavy	17	20.76 ± 9.71			0.13
Somewhat heavy	26	18.77 ± 8.52			
Average	45	16.16 ± 7.68			
**Father**						
**Employment status**						
Employed	71	17.86 ± 8.54			0.93
Unemployed/unpaid leave/unknown	17	17.65 ± 8.31			
**Work stress levels**						
Very heavy	12	22.25 ± 6.57			0.15
Somewhat heavy	34	17.03 ± 9.14			
Average	42	17.19 ± 8.13			
**Education**						
< College degree	46	17.67 ± 8.58			0.87
≥ College degree	42	17.98 ± 8.41			
**Mother**						
**Employment status**						
Employed	41	15.15 ± 7.62			0.005*
Unemployed/unpaid leave/unknown	47	20.15 ± 8.53			
**Work stress levels**						
Very heavy	8	21.50 ± 9.27			0.06
Somewhat heavy	17	13.82 ± 7.67			
Average	63	18.43 ± 8.30			
**Education**						
< College degree	44	18.02 ± 8.65			0.82
≥ College degree	44	17.61 ± 8.34			

Abbreviation: SD, standard deviation.

**Table 2:** C‐POS responses by group.

	PaPaS Scale score		*p*‐value	
	>=20 (n = 48)	15‐19 (n = 40)		
C‐POS item	Mean ± SD	Mean ± SD		SMD
Item 1 Pain	**1.65** ± **1.71**	**0.85** ± **1.35**	0.02*	**0.51**
Item 2 Other body problems	1.88 ± 1.92	1.38 ± 1.71	0.20	0.27
Item 3 Feeding	0.98 ± 1.23	0.83 ± 1.24	0.56	0.12
Item 4 Cried	**1.35** ± **1.68**	**0.80** ± **1.38**	0.04*	**0.35**
Item 5 Felt happy	2.46 ± 1.77	1.80 ± 1.76	0.09	0.37
Item 6 Playing	**2.19** ± **1.67**	**1.43** ± **1.88**	0.047*	**0.43**
Item 7 Questions answered	0.73 ± 1.53	0.55 ± 1.38	0.57	0.12
Item 8 Worry	4.29 ± 1.24	3.98 ± 1.40	0.26	0.23
Item 9 Shared own feelings	**1.65** ± **1.85**	**0.75** ± **1.30**	0.01*	**0.55**
Item 10 Received adequate information	0.94 ± 1.10	0.65 ± 0.92	0.19	0.28
Item 11 Received enough help and advice	1.38 ± 1.42	1.00 ± 1.20	0.19	0.28
Item 12 Family confidence	0.98 ± 1.38	0.78 ± 0.83	0.39	0.17

Abbreviations: SD, standard deviation; SMD, standardised mean difference.

### 3.2 Care Needs Identified From Mixed‐Methods Analysis

A total of 28 interviews were conducted with 12 family caregivers and 14 healthcare professionals. Professionals’ age ranged from 27 to 56 years, with a median age of 36 years, and caregivers’ age ranged from 33 to 55 years, with a median age of 51 years (Table 3). Most interviews were conducted in person, with 10 (35.7%) conducted via video calls due to COVID‐19.

**Table 3:** Characteristics of healthcare professionals and family caregivers interviewed.

Characteristics	Healthcare professionals (*n* = 14)	Family caregivers (*n* = 12)	Ill children and young adults of family caregivers interviewed (*n* = 13)
**Median age at the time of interview, years (range)**	36 (27‐56)	51 (33‐55)	16 (1‐28)
**Gender, n (%)**			
Female	11 (78.6)	8 (88.9)	
**Education, n (%)**			
None	0	0	3
Special education	0	0	2
Preschool	0	0	1
Elementary school	0	1	2
Junior high school	0	0	1
High school	0	5	3
College	7	6	1
Masters and above	7	0	0
**Type of occupation, n (%)**	Nurse [5 (35.7)]	Professionals [2 (16.7)]	None [11 (92.3)]
	Case manager [1 (7.1)]	Services and sales workers [3 (25.0)]	Professional [1 (7.7)]
	Shared care nurse [1 (7.1)]	Elementary occupants [1 (8.3)]	Small‐scale community workshops [1 (7.7)]
	Head nurse [2 (14.3)]	Housewife [6 (50.0)]	
	Paediatrician [2 (14.3)]		
	Palliative care specialist [1 (7.1)]		
	Psychologist [1 (7.1)]		
	Chaplain [1 (7.1)]		
**Marital status, n (%)**			
Married	5 (35.7)	10 (83.3)	0
Separated/divorced/single	9 (64.3)	2 (16.7)	13 (100.0)
**Number of children per families, n (%)**			
0	9 (64.3)	0	
1	2 (14.3)	5 (41.7)	
2	3 (21.4)	4 (33.3)	
3	0	2 (16.7)	
4	0	1 (8.3)	
**Religion, n (%)**			
Atheist	9 (64.3)	2 (16.7)	
Folk beliefs	0	3 (25.0)	
Buddhism	2 (14.3)	5 (41.7)	
Taoism	0	1 (8.3)	
Christianity	3 (21.4)	1 (8.3)	
**Diagnostic group, n (%)**			
Oncology			5 (38.5)
Neurology			2 (15.4)
Circulatory			1 (7.7)
Gastrointestinal			1 (7.7)
Metabolic			3 (23.1)
Congenital			1 (7.7)

### 3.3 Seeking Comprehensive Care Guidance as Care Needs Evolved

Families often seek professional guidance on home medical care, symptom management, nutrition, psychosocial well‐being, and end‐of‐life care. Managing problems such as poor appetite, ascites, and osteoporosis and the use of nutritional supplements is particularly challenging. Limited resources and uncertainties about illness progression often lead families to rely on ‘trial‐and‐error approaches.’ Children often have difficulty expressing their needs; thus, families must be ‘proactive and observant.’ For instance, one mother mistakenly thought that her son's irritability was due to constipation when it was actually caused by toothache.

I didn't know I had to prepare a spare one [nasogastric tube] at home, and I had little experience [using the tube]…… I went to the hospital to learn how to use a suction catheter, and then a nasogastric tube as well.

(Mother 3)

The quantitative findings complemented these accounts, showing that children and young adults with greater normative palliative care needs had poorer outcomes for pain and crying (Table 2). These patterns were consistent with the caregivers’ descriptions of difficulties in recognising children's discomfort, responding to behavioural changes, and supporting their emotional and social needs.

Families also seek guidance on communicating with children about their illnesses and supporting them in peer relationships, including experiences of bullying at school. Those receiving comprehensive care guidance typically exhibit two attitudes: active and passive recipients. Active recipients actively seek information and are ‘willing to do everything possible’ for their child, often ‘having considered all aspects of the child's illness.’ They find ‘shared experiences’ from others helpful. In contrast, passive recipients often struggle to cope, described as those ‘could not get over it’, avoiding social activities and guidance. Healthcare professionals viewed providing guidance as a way to encourage families to engage in more physical touch, foster ‘heart‐to‐heart communication’ with their child, and ‘create memories.’ Moreover, they are expected to ‘empathise with families’ situations’ and ‘understand their barriers.’ Together, these findings suggest that care guidance should address not only clinical procedures but also symptom recognition, emotional expression, and participation in everyday activities.

### 3.4 Integrating Care Plans Into Everyday Family Routines

Caring for a child—including providing medical attention, rehabilitation, and education—can disrupt the family's routine. Families value care plans that are ‘organised by the whole team’, allowing them to ‘follow it step by step.’ Such plans help families transition from ‘fearing that the child might get out of control’ to managing their child's treatment effectively.

Quantitatively, caregivers reported relatively few difficulties in receiving adequate information, help, and advice. Access to information, help, and advice did not significantly differ between the PaPaS groups (Table 2). The qualitative findings added context by showing that the usefulness of support depended not only on its availability but also on whether professional recommendations could be translated into practical plans that fit family routines.

Family routines vary according to their values and structure. Chaotic surroundings often challenge families in managing treatments and complications. For instance, a paediatric nurse described a parent who anxiously monitored a child with neurological conditions 24/7. Families in rural areas may relocate for treatment, believing that they can find a better chance of being cured, causing further disruption. Despite these disruptions, there is hope that children ‘live as normally as possible.’ For many, a ‘normal kid’ enjoys a good appetite, moves independently, accesses education, socialises with peers, and ‘experiences happiness.’ Quantitatively, children and young adults with greater normative palliative care needs reported poorer outcomes related to playing and a nonsignificant tendency toward poorer outcomes for feeling happy (Table 2). These findings complemented caregivers’ accounts, which suggested that increasing symptoms and care demands could disrupt children's participation in and enjoyment of everyday family and social life.

She [the mother] does not consider her son to be ‘abnormal’. …… She doesn't think he has seizures or anything that should be medically managed, just treating him like a child who easily catches colds or is fragile.

(Paediatric nurse 2)

Caregivers also performed the necessary work to enable children and young adults to participate in schools or workplaces. Bedside itinerant teachers help children ‘learn like their peers’ while maintaining daily structure. Some family caregivers noted that their child's long‐term illness led to bad temper and undisciplined behaviour. As illness became the focus of their lives, families felt that expectations for their children sometimes became ‘meaningless’ and struggled to understand that ‘some compromises had to be made.’ Case managers assist parents in ‘recognising priorities’, such as valuing school attendance for social development rather than focusing primarily on academic achievement.

### 3.5 Sharing Enduring Caregiving Responsibilities Over Time

Long‐term caregiving requires long‐term specialised knowledge and skills and places substantial emotional distress on caregivers. Professionals often recognise mothers’ need for emotional support, but other family members might be unprepared to provide it. An obstetrics and gynaecologist nurse noted that fathers are ‘pressured to be strong’ for mothers who experienced stillbirth, leaving them without emotional care. Half of the participating families experienced financial difficulties because caregiving interrupted employment; flexible schedules and part‐time work helped some caregivers balance their responsibilities.

Caregivers of children and young adults with greater normative palliative care needs reported greater difficulty sharing their feelings, whereas caregiver worry was high across both PaPaS groups (Table 2). Qualitative accounts helped explain these patterns: caregivers described persistent anxiety and pressure but often suppressed their emotions in front of the child.

I think those who take care of such ill children for a long time …… The anxiety persists over an extended period, and it couples with an invisible pressure…… Thus, your emotion becomes unstable, yet you can't show it in front of the child.

(Aunt 8)

Families seek assistance to ‘improve their daily lives’ by relying on formal and informal support for their ill children. Formal support includes professional services, schools, residential facilities, and respite care, which offers crucial breaks for caregivers. However, some reported that teachers may refuse to accept children and young adults with serious long‐term conditions or ask parents to accompany them in class because of their high medical or attentive needs. Informal support often came from relatives, friends, and the caregivers of other hospitalised children, who helped manage family businesses, care for healthy siblings, or provide mutual assistance during hospital stays, creating what caregivers described as ‘a circle of goodness.’ Some families emphasise teaching caregiving skills to healthy siblings to ‘foster a strong bond’, acknowledging the uncertain lifespan of the ill child and the fact that ‘parents will not be around forever.’ Healthcare professionals suggested a collaborative approach involving multiple professionals, similar to a ‘relay race’, and emphasised the importance of ‘compassionate support’ that respects families’ decisions and ‘allows them to choose or refuse care.’

I used to feel exhausted when I got home, but still had to help him [son] shower……I need to work on Saturday, and the home carer can take him to the rehabilitation center…… It truly makes a difference to have that support.

(Mother 7)

Although families wanted others to share the caregiving burden, some understood their responsibility as a source of meaning. One mother with a child facing cancer and Down syndrome described her son as a ‘gift from God’ and viewed caring for him as a blessing rather than a burden. However, attitudes toward sharing care were not uniform. One participant viewed children in residential facilities negatively, illustrating the social and emotional barriers that could prevent families from transferring caregiving responsibilities to others.

Parents often find it challenging to let go of their children. They carry their children by their side and raise them with great love. In contrast, those who do let go throw their children to residential facilities.

(Paediatric nurse 2)

Together, these findings indicate that families with greater professionally assessed palliative care needs may experience more difficulty expressing their emotions, reinforcing the importance of sustained support that allows caregiving responsibilities and emotional burdens to be shared over time.

### 3.6 Balancing Living in the Present With Preparing for the Future

Caregivers have varied perspectives on time orientation regarding their child's illness. Those whose children had recently experienced a life‐threatening event or were approaching the end of life often ‘focused on the present’ and prioritised ‘basic survival needs.’ Because ‘nothing can be taken for granted’, future planning could seem ‘unrealistic’ or like a ‘fantasy’, leading families to ‘take it 1 day at a time’ and show limited interest in advance care planning.

Quantitatively, family confidence did not differ significantly between the PaPaS groups (Table 2). The qualitative findings provided further context by showing that confidence in caring for the child was not determined solely by the severity of palliative care needs. Rather, it developed through caregiving experiences, access to information, recognition of positive changes, and the ability to adapt to expectations while maintaining hope.

Although some healthcare professionals believed that children and young adults with serious long‐term conditions might ‘have no future’, families continued to hope and often ‘prioritise long‐term care over palliative care.’ They drew reassurance from small positive developments, such as ‘At least he is not too difficult to care for’ or ‘At least he said he is okay after having severe vomiting.’ Many believed that maintaining a ‘positive attitude’ and ‘being willing to meet the child's needs’ were more important than the caregiving environment or severity of illness.

Approaches to the future also varied. Some families sought to prevent deterioration through rehabilitation, whereas others travelled with their child ‘in case it becomes too late.’ Families considered the child's dignity and quality of life in relation to physical independence and family attitudes. One mother emphasised that care provided ‘merely out of duty’, without considering ‘how the child is doing today’ or ‘how he feels’, could undermine the child's dignity and eventually lead the family to ‘let go.’ Clear information about prognosis and the potential benefits and harms of treatment was therefore important for decision‐making and avoiding regret.

Healthcare professionals emphasised respecting families’ readiness to consider future losses rather than attempting to change their temporal orientation prematurely.

……[respect] her mindset at that moment. If I insist on hitting her down, when her mindset is not at the level of accepting the loss, she will hate me.

(Clinical psychologist 10)

After their child's death, some caregivers extended their accumulated experience to the wider community by participating in parent groups and volunteer work. Supporting other families gave continuous meaning to their caregiving experiences. Together, these findings suggest that family confidence was not directly associated with the level of normative palliative care needs; rather, caregivers sustained confidence by drawing on experience, information, hope, and a present‐focused approach while gradually preparing for an uncertain future.

### 3.7 Integrated Interpretation

Across the four themes, the qualitative findings extended the quantitative assessment of palliative care needs by revealing how these needs were experienced and managed within everyday family life. Although caregivers generally reported receiving adequate information and support, the qualitative findings showed that support was most useful when it could be translated into coordinated and practical care plans that fitted family routines. The qualitative findings also added a temporal dimension that was not captured by the quantitative measures, showing how caregivers balanced living in the present with preparing for an uncertain future. Taken together, the integration suggests that understanding palliative care needs requires attention not only to the level of need identified through standardised assessment, but also to how families interpret, manage, and adapt to these needs within their everyday lives and while balancing present caregiving demands with an uncertain future.

## 4 Discussion

This study integrated professionally assessed palliative care needs, patient‐ or proxy‐reported outcomes, and caregivers’ accounts of everyday care. Children and young adults with higher normative palliative care needs had poorer C‐POS outcomes related to pain, crying, and playing, and their caregivers reported greater difficulty sharing their feelings. The standardised effect sizes for these statistically significant item‐level differences were small to moderate. However, the clinical importance of these item‐level differences remains uncertain because the available minimum important differences for the C‐POS has been established for the total scores rather than for individual items [19]. The qualitative findings contextualised the potential relevance of these differences by showing that caregivers often struggled to recognise the sources of their child's distress, relied on trial‐and‐error approaches to symptom management, and suppressed their emotions while providing care. Together, the quantitative and qualitative findings indicate that greater professionally assessed palliative care needs were associated with poorer symptom‐related and participation outcomes and greater difficulty in caregivers’ emotional communication. This interpretation is consistent with previous evidence linking children's symptom burden with psychological distress among parents of children receiving palliative care [20]. Therefore, children and young adults with higher PaPaS scores may require comprehensive support encompassing symptom recognition and management, psychosocial assessment, and opportunities for caregivers to discuss their concerns.

The omega coefficients observed for the child‐related and caregiver‐related items in this study (0.72 and 0.62, respectively) were broadly consistent with previous C‐POS validation findings, which reported omega coefficients of 0.67–0.71 for the child subscale and 0.49 for the family subscale [19]. Given that palliative care outcomes encompass heterogeneous physical, emotional, and psychosocial experiences, lower internal consistency may be expected for multidimensional measures and does not necessarily indicate inadequate reliability [19].

The integrated findings clarified why the availability of information and support may be insufficient. Although caregivers reported relatively few difficulties in obtaining information, help, and advice, the qualitative findings indicated that support was most useful when recommendations were translated into coordinated and practical care plans that could be incorporated into family routines. Caregivers coordinated medical care, rehabilitation, education, employment, and sibling care while adapting to changing symptoms and uncertain illness trajectories. The association between higher PaPaS scores and poorer playing outcomes was consistent with caregivers’ accounts that increasing symptoms and care demands could disrupt children's participation in ordinary family, school, and social activities. Similarly, paediatric palliative care standards emphasise interdisciplinary care coordination across healthcare and community settings and attention to the physical, psychosocial, and communication needs of children and families [21]. These findings suggest that care coordination should extend beyond providing services or information: families may benefit from a designated professional who can integrate recommendations across settings, clarify priorities, and adapt care plans as their circumstances evolve.

Caregivers of children and young adults with higher PaPaS scores reported greater difficulty sharing their feelings. The qualitative accounts helped explain this finding by describing prolonged anxiety, emotional suppression, and pressure to remain strong for the child. Families sustained caregiving through a ‘relay race’ involving relatives, friends, other families, schools, healthcare professionals, respite services, and residential care. However, the acceptability and availability of support varied. Some caregivers found that sharing responsibilities enabled them to continue working and caring for other family members, whereas others perceived transferring care as inconsistent with parental love or responsibility. In addition, previous research has identified child symptom burden, financial difficulties, and limited respite availability as contributors to parental distress [20, 22]. Therefore, emotional and practical support should be tailored to families’ circumstances and readiness to share or accept caregiving assistance.

Maternal unemployment was associated with poorer C‐POS outcomes. However, this association should not be interpreted as evidence that unemployment adversely affects care outcomes. Reverse causation is possible because mothers may have left employment in response to their child's illness severity or caregiving demands. Residual confounding by socioeconomic disadvantage, illness complexity, and access to support services should also be considered. The qualitative findings supported these possible explanations by illustrating how prolonged caregiving interrupted employment and intensified financial and emotional pressures. Similarly, previous studies found that caring for a child with a life‐limiting condition may require parents to reduce their working hours or leave employment, thereby increasing families’ financial vulnerability [20, 23]. Although family caregiving expectations in Taiwan may also be relevant, cultural mechanisms were not directly examined in this study; therefore, this interpretation should remain tentative. Flexible work arrangements, financial assistance, and respite services may help families sustain employment and share caregiving responsibilities [22, 24].

This study highlights the dynamic nature of care needs and the different time orientations through which caregivers approached their child's care. Some caregivers adopted a present‐oriented perspective, focusing on immediate survival, symptom management, and maintaining everyday routines. Others were more future‐oriented, emphasising rehabilitation, prevention of deterioration, meaningful experiences, and preparation for future care and end‐of‐life decisions. These orientations were not fixed or mutually exclusive; rather, caregivers moved between living in the present and preparing for the future as the child's condition, perceived prognosis, and family circumstances changed.

Family confidence did not differ significantly between caregivers of children and young adults with PaPaS scores ≥ 20 and those with scores of 15–19. The qualitative findings contextualised this result by suggesting that confidence was not determined solely by the level of normative palliative care needs. Instead, caregivers sustained confidence through accumulated experience, access to information, hope, and the ability to adjust their time orientation in response to uncertainty. A present‐oriented perspective helped some caregivers manage immediate demands, while gradually developing a future‐oriented perspective enabled others to consider rehabilitation, meaningful family experiences, advance care planning, and possible deterioration.

Recognising caregivers’ time orientation may help healthcare professionals provide support consistent with families’ current priorities and readiness. Introducing advance care planning when caregivers remain focused on immediate survival may be perceived as premature and may create conflict. However, an exclusively present‐focused approach may also delay discussions that can help families prepare for future decisions. Therefore, paediatric advance care planning should be approached as an iterative process, with the timing and content of discussions adapted to parental readiness and revisited as the child's condition and the family's time orientation change [23, 25, 26].

Taken together, these findings support a stepped referral pathway in which increasing PaPaS scores, worsening symptoms or emotional distress, reduced participation in everyday activities, and caregiver communication difficulties prompt timely action before a crisis occurs. Previous reviews have identified symptoms, psychosocial and emotional concerns, communication difficulties, and care‐management needs as relevant criteria for paediatric palliative care referral [27]. Thus, screening should be linked to actionable responses, including interdisciplinary review, symptom‐management guidance, a designated care coordinator, caregiver education and emotional support, community and respite services, and timely consultation with paediatric palliative care specialists.

### 4.1 The Strengths and Limitations of the Study

This study integrated professional assessments, patient‐ or proxy‐reported outcomes, and caregivers’ experiences across three medical centres in Taiwan. However, the cross‐sectional design precluded causal inference, and the modest sample size prevented multivariable adjustment. Recruitment from tertiary medical centres may have introduced selection bias. Only one father was interviewed, which limited insight into fathers’ experiences. Finally, the findings may not be generalisable beyond Taiwan because of the differences in healthcare systems and sociocultural contexts.

## 5 Conclusion

By integrating professionally assessed needs, patient‐ or proxy‐reported outcomes, and caregivers’ experiences, this study provided a comprehensive understanding of the care needs of children and young adults with serious long‐term conditions prior to referral to specialist palliative care. The findings highlight the need for coordinated child‐ and family‐centred support that responds to evolving symptoms, everyday family routines, caregiver burden, and emotional communication. Healthcare professionals should also recognise caregivers’ changing time orientations and provide support that addresses immediate priorities while gradually preparing families for future care and decision‐making.

## Author Contributions

**Shih‐Chun Lin:** conceptualisation, writing – original draft, writing – review and editing; funding acquisition, methodology, data curation, formal analysis, investigation. **Mei‐Chih Huang:** conceptualisation, methodology, writing – review and editing, resources, supervision. **Hsiang‐Ying Lu:** investigation, project administration, resources, data curation, writing – review and editing. **Ying‐Chu Chen:** investigation, data curation, writing – review and editing. **Chao‐Neng Cheng:** data curation, writing – review and editing. **Frank Leigh Lu:** data curation, writing – review and editing. **Jong‐Hau Hsu:** resources, writing – review and editing.

## Ethics Statement

The study was approved by the Kaohsiung Medical University Hospital (KMUHIRB‐SV(I)−20220087), National Cheng Kung University Hospital (B‐BR‐110‐012), and the National Taiwan University Institutional Review Board (202202028RINA).

## Conflicts of Interest

The authors declare no conflicts of interest.

## Data Availability

The datasets used and/or analysed during the current study are available from the corresponding author on reasonable request.
